# The Novel iMPACT Tool and Quadrant Protocol for Peri-Implantitis: Surface Refinement and Re-Osseointegration Validated by SEM/EDS and Long-Term Clinical Case Reports

**DOI:** 10.3390/medicina61061094

**Published:** 2025-06-16

**Authors:** Gustavo Vicentis Oliveira Fernandes, Bruno Gomes dos Santos Martins, Juliana Campos Hasse Fernandes, Yankel Gabet, Amiram Vizanski

**Affiliations:** 1 Missouri School of Dentistry & Oral Health, A.T. Still University, 1500 Park Dr., St. Louis, MO 63104, USA; 2 GF10Foundation, St. Louis, MO 63104, USA; 3 Doctoral Program “Surgery and Dentistry”, Department of Surgery, University of Salamanca, 37007 Salamanca, Spain; 4 Department of Anatomy and Anthropology, Faculty of Medical and Health Sciences, Tel Aviv University, Tel Aviv-Yafo 6997801, Israel; 5 Private Practice, Tel Aviv-Yafo 6688218, Israel

**Keywords:** peri-implantitis, dental implants, bone resorption, surgical procedures, device, clinical protocol

## Abstract

*Background and Objectives*: The goal of this study was to introduce a novel device, the iMPACT implant planer, designed to machine (create a complete smooth surface) contaminated implant surfaces intraorally, promoting peri-implant tissue healing and possible re-osseointegration, and the new Quadrant protocol, evaluating them in vitro and clinically. The null hypothesis was that there would be no improvement in the clinical parameters for the implants with peri-implantitis (PI) treated with the new protocol and tool. *Materials and Methods*: The Quadrant protocol was used in conjunction with the iMPACT tool, which primarily functions to remove biofilm and microbial contaminants from the exposed implant surface, while simultaneously preparing the surface through standardized implantoplasty, thereby enhancing the potential for re-osseointegration. An in vitro analysis was developed, and three medium/long-term cases were presented, detailing the procedures and outcomes. *Results*: The in vitro assessment showed smooth surfaces after treatment. Different areas presented minimal particles (<1 μm) on the implant surface, with a high content of titanium (Ti) and tungsten (W). In case 1, severe and advanced peri-implantitis around implants #46 and #47 was found. A combination of resective (Quadrant + iMPACT) and regenerative surgery was used for treatment, along with a buccal single flap (BSF). Significant clinical and radiographic improvements were observed at 14 and 43 months postoperatively, including vertical bone gain with re-osseointegration and stable probing depths (PDs). In the second case, a severe PI and prosthesis instability were observed. Resective (Quadrant + iMPACT) and regenerative procedures were applied. At 3 and 12 months postoperatively, clinical and radiographic evaluations demonstrated significant improvements with re-osseointegration, including PDs reduced to 0–1 mm and a vertical bone gain of approximately 6.5 mm. In case 3, mandibular implants from 42 to 47 exhibited inflammation, suppuration, and moderate-to-severe bone loss. Just resective surgery (Quadrant + iMPACT), without grafting, was performed. At 6- and 12-month follow-ups, clinical and radiographic assessments showed the resolution of inflammation, stable bone levels, and healthy peri-implant gingiva. *Conclusions*: Favorable outcomes were achieved using the iMPACT and Quadrant protocols in the three clinical cases, resulting in re-osseointegration when combined with regenerative procedures. The favorable medium/long-term outcomes achieved, despite the patient’s complex medical history and, at times, inconsistent oral hygiene, underscore the potential efficacy of such interventions.

## 1. Introduction

In the 2017 World Workshop for the Classification of Periodontal and Peri-Implant Diseases (PID) and conditions, peri-implantitis (PI) was defined as a chronic inflammatory state around a dental implant, characterized by tissue destruction and microbial action as a primary driving factor [1,2,3,4,5]. PI’s characteristics include edema, bleeding on probing (BoP), and/or suppuration, affecting the bone section, which leads to progressive bone loss and, ultimately, implant loss if left untreated. PI progresses faster than periodontitis, being more frequently found on a hastened, unsteady pattern [3,6,7,8]. Chronic plaque accumulation on the implant is strongly associated with disease development and progression [3,9]. It appears that a microbiome alteration can be observed in peri-implantitis sites, leading to the hypothesis that a highly specific microbiological environment is established, which facilitates the progression of the disease [8,10,11,12]. It is valid to highlight that the volume of peri-implant soft tissues, as well as the thickness of keratinized and connective tissue, can influence the long-term stability of the peri-implant mucosal seal, being a cause or cofactor of peri-implantitis and a possible therapy to be considered [13].

Several methods have been thoroughly developed and studied, considering the challenge of treating PI [1,6,8,14]. Before treatment, the diagnosis of the disease should be as accurate as possible; peri-implant mucositis (PIM) displays one or more BoP sites, an abundant blood line arising from the peri-implant sulcus, or suppuration at gentle probing. No bone loss should be present (except for initial bone remodeling). PI shows BoP or suppuration after probing, increased probing depth (PD) ≥ 6 mm, and radiographic bone loss [6,8].

As knowledge of PID increases, clinical guidelines have been developed to aid in the treatment of PI, as well as its prevention [15]. PID treatment should always start with a nonsurgical approach (NS). This treatment mode encapsulates the supra- and subgingival cleaning of the bacterial biofilm present. This can be achieved using manual or ultrasonic methods, with or without the aid of adjunctive agents (i.e., local antibiotics [ABs], systemic ABs, local irrigants such as chlorhexidine, and laser application); however, the benefit of these approaches is not yet fully clarified [1,6,8,14].

The successful integration and retention of dental implants are influenced by various factors that must be adequately addressed to minimize the risk of PI and implant loss. Among these factors, initial implant stability, positioning relative to anatomical parameters, and surgical protocols play critical roles [16]. Initial stability is paramount for implant success. It is primarily influenced by the bone-to-implant contact (BIC) ratio and the density of the surrounding bone. In addition, the biomechanical properties of the implant design, including its geometry and surface characteristics, have a significant impact on osseointegration [15]. Moreover, the positioning of implants also has significant implications [17]. Critical parameters include the available interradicular space and bucco-lingual dimensions, ensuring at least 2 mm of buccal bone anterior to the implant fixture and maintaining the implant platform 1.5 to 3 mm apical to the cementoenamel junction (CEJ) of adjacent teeth, which can facilitate both functional and aesthetic outcomes, while reducing the risk of complications, such as crestal bone loss [18]. Surgical protocols also play a vital role; for example, flapless techniques may enhance postoperative healing and minimize damage to soft tissues, thereby promoting better peri-implant conditions [19]. Furthermore, the anatomical location of the implant affects survival rates, with studies indicating that implants placed in the posterior maxilla face a higher risk of failure compared to those placed in other sites [20]. Bone quality and density, as well as proximity to vital anatomical structures, must be taken into consideration when planning implant placement in these regions. Additionally, factors such as implant diameter have been reported to correlate with failure rates inconsistently across various studies [21].

Depending on the host’s response, which involves microbial profile and biofilm formation; immune response involving various cellular components, particularly the activation of macrophages and the accumulation of inflammatory cytokines; genetic factors and preexisting conditions, such as IL-1 gene polymorphisms; tissue characteristics, such as the lower vascularity and cellularity of peri-implant connective tissue; and the level of PID progression and stage, NS treatment appears sufficient for treating only peri-implant mucositis (PIM) but does not arrest PI progression. In that case, a surgical approach (SA) is recommended. This surgical intervention involves elevating a flap to properly decontaminate the implant’s surface [6,8,12,22,23].

SA can be divided into the following three methods: (1) resective or non-reconstructive, (2) reconstructive, and (3) a combination of methods (i.e., implant decontamination with implantoplasty and reconstructive surgery) [6]. Non-reconstructive approaches appear to yield clinical improvements; however, disease recurrence was observed in nearly 50% of cases after 5 years. It is hypothesized that the increased soft tissue recession caused by the resective surgical approach is one of the factors contributing to late disease recurrence. Bone defect morphology is also an important factor [6]. In reconstructive approaches, the goal is to restore lost bone, prevent soft tissue recession, and achieve new implant osseointegration. Recent studies have shown that reconstructive methods retained an approximately 55% success rate at 5 to 7 years post-intervention. Outcomes may vary significantly due to the initial bone defect [1,6,22].

The efficacy of implant surface decontamination has been a key topic of discussion, as it is a crucial step in reducing bacterial load and eliminating the primary component for PID establishment and progression. Implantoplasty, titanium brushes, and ultrasonic devices have shown some degree of improvement over other methods. Implantoplasty involves rotating burs at the implant surface level, modifying the mechanical characteristics of the surface, and creating a smoother, non-plaque-retaining ambiance [1,6,8,24]. The procedure may include the removal of implant threads at a supra-bone level. Rosen and Tarnow [25] raised the question of whether subcrestal implantoplasty could be performed and showed a literature-first case with some promising regenerative potential.

In combined interventions, implantoplasty showed a promising benefit compared to flap debridement [14]. A 3-year randomized controlled trial (RCT) demonstrated greater PD reduction, reduced inflammation of the surrounding tissues, and a stable marginal bone level [6]; in agreement with these results, similar reports have been found [26,27]. Following the authors’ statement [6], treatment successes were defined as maintaining implant function after applying the combined intervention of SA and implantoplasty, with results achieving 97.5% and 94.7% at 6 and 24 months, respectively. Moreover, a systematic review demonstrated that implantoplasty could significantly improve these clinical markers, showing a mean reduction in PD ranging from approximately 2.1 mm to 2.3 mm over a follow-up period of one to two years [28,29,30]. While implantoplasty appears to be a promising decontamination method, some concerns have been raised regarding titanium (Ti) particle scattering and biomechanical alterations in implant behavior [6,31,32]. In an in vitro study, rubber dam and bone wax appeared to reduce the number of Ti particles significantly scattered [33]. Another in vitro analysis stated that the particles released from implantoplasty induced a pro-inflammatory response and decreased the expression of osteogenic biomarkers [34]. The biomechanical concern arising from implantoplasty appears to be relevant only in reduced-diameter implants (3.3 to 3.75 mm) [35], as standard-diameter implants (4.1 to 4.7 mm) seem to retain their mechanical properties [6].

Then, implantoplasty appears to be the most promising surface decontamination method, with favorable results having been achieved with subcrestal implantoplasty, and micro- and nanometric particles are detrimental to achieving an adequate environment. Additionally, a process known as re-osseointegration of the implant surface after implantoplasty has emerged, involving several critical factors. Studies demonstrate that high re-osseointegration rates can be achieved, particularly when employing adjunctive treatments, such as autogenous bone grafts or synthetic hydroxyapatite [36,37]. In a pre-clinical study, significant re-osseointegration was observed at 6 months post-treatment, with more favorable outcomes associated with implants featuring rough surfaces compared to those with smoother surfaces, indicating that surface texture plays a vital role in enhancing re-osseointegration after intervention [38]. Otherwise, caution is advised, as the anatomical limitations of decontaminated surfaces may restrict complete re-osseointegration at defective sites, particularly on rough surfaces where inflammation may still be present [39].

Hence, this study aimed to introduce an innovative methodology (Quadrant protocol) and device (iMPACT) to treat peri-implantitis cases. The rationale of this case series was to demonstrate a standardized approach (protocol and tool) for treating peri-implantitis, promoting faster implant surface smoothness, eliminating the disease, and enhancing the quality of the local tissue. The null hypothesis was that there would be no improvement in the clinical parameters for the implants with PI treated with the new protocol and tool.

## 2. Materials and Methods

This prospective cohort case series was conducted in accordance with the Declaration of Helsinki (1964, updated 2024) and was approved by the local Institutional Review Board (IRB) (n. 0009458-2, Tel Aviv University, Tel Aviv-Yafo, Israel). Three patients with peri-implantitis and a follow-up period of at least 12 months were included in this case report. The patients were managed and supervised by a highly experienced dentist (A.V.). For the first time in the literature, the iMPACT^®^ tool (Tel Aviv-Yafo, Israel) and Quadrant protocol are being presented. All patients were thoroughly informed in detail about the treatment plan, expected outcome, procedures, risks and benefits, and treatment options, which were meticulously explained. The patients signed the consent form and agreed to the instructions.

## 3. iMPACT Device

**Figure 1:**
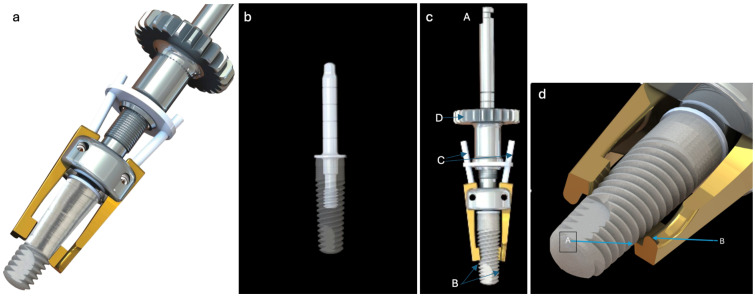
(**a**) iMPACT tool (new tool) to treat peri-implantitis through implantoplasty; (**b**) pin for connection and stabilization of the tool screwed in the dental implant; (**c**) (**A**) the cutting tool’s rotational motion, (**B**) preparing the area for treatment, (**C**) springs positioned above the working area, (**D**) fine-tuning the cutting depth and blade position is achieved using an adjustment nut; (**d**) detailed illustration showing the iMPACT’s blade in position for a standardized implantoplasty. The iMPACT, featuring the precision cutting blades, was used for simultaneous implantoplasty and controlled bone modification.

The iMPACT device is a precision-engineered, three-axis lathe machine that enables accurate circumferential decontamination of the implant surface (Figure 1). It employs a centric borehole mechanism to control cutting blade movements, ensuring the generation of large particles, while maintaining surface smoothness. It comprises two interdependent components that work harmoniously to deliver precise surgical action, as follows: (1) The hinge (Figure 1b) acts as the structural foundation for guiding the cutting tool with accuracy, maintaining alignment along the implant’s long axis to ensure uniform treatment. It is threaded into the internal threads of the implant at the abutment connection area. This attachment extends the implant’s long axis, providing a stable and precise path for the cutting tool to follow rotationally and vertically. (2) The cutting tool (Figure 1d) removes titanium layers, smooths the implant surface, and induces bone healing reactions. In addition, it provides rotational and vertical motion to access and treat the implant surface comprehensively. It is inserted through a central borehole on the hinge. Its design enables free rotation around the hinge, while allowing vertical motion along the implant’s axis.

## 4. Quadrant Protocol for Peri-Implantitis Treatment (Implantoplasty)

The Quadrant protocol is an innovative, evidence-based solution, and comprehensive protocol for treating peri-implantitis. It uses a specialized tool (iMPACT) and was developed scientifically, ensuring a thorough approach to managing PI (standardized implantoplasty). The protocol involves four essential steps, each addressing critical aspects of PI treatment, including (1) decontamination, (2) bone healing, (3) infection prevention, and (4) re-osseointegration.

**Figure 2:**
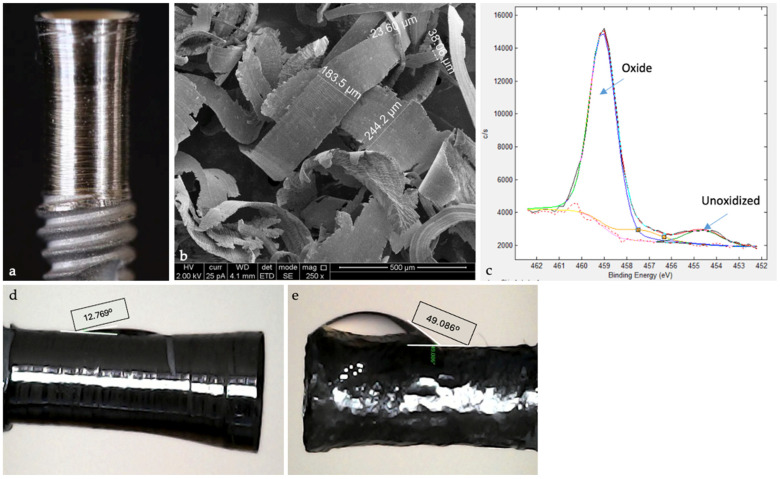
(**a**) The contaminated implant’s layer was removed meticulously under controlled conditions, with power control and accurate cutting, to achieve a smooth surface. (**b**) Large titanium particles generated during the process are easily detectable clinically. (**c**) New implant surface with an area average roughness (Sa) value of ~0.5 µm–binding energy of 459 eV; (**d**) The restored surface exhibits increased hydrophilicity using iMPACT (water drop angle 12.769°); (**e**) Compared to traditional methods of implantoplasty (tungsten bur—water drop angle 49.086°).

**Figure 3:**
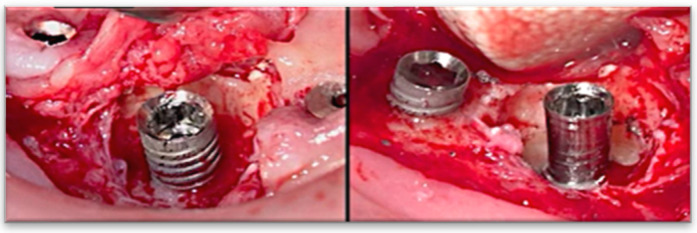
Applying the Quadrant protocol, the iMPACT tool was utilized to treat a case of peri-implantitis. After using iMPACT, the new implant surface was machined to be smooth.

Step 1. Removing the contaminated layer and restoring the passivation layer: The focus is on removing the contaminated layer from the implant surface, while re-establishing a stable oxide layer to enhance implant biocompatibility and stability. The contaminated layer is removed meticulously under controlled conditions, power control, and accurate cutting (Figure 2a). Unlike conventional implantoplasty methods that use irrigation, this process relies on consistent suction airflow to cool the site and remove debris. The cutting approach prevents nanoparticle production compared to the conventional abrading method, as nanoparticles are more biologically reactive and can exacerbate inflammation [40,41]. The removal process generates large titanium particles (Figure 2b), which are visually detectable and easily removed, thereby reducing the inflammatory burden [42]. Smaller nanoparticles are not expected to be formed using the Quadrant protocol, as it provokes more severe macrophage-driven reactions [43]. The restored surface exhibits increased hydrophilicity (Figure 2c–d) compared to traditional methods of implantoplasty (using burs), which can enhance protein absorption, cell attachment, and osseointegration. Hydrophilic surfaces are critical for promoting favorable biological responses and rapid healing [44]. Then, the natural affinity of titanium exposed to oxygen allows for the spontaneous formation of a stable oxide layer, which enhances implant surface stability and biocompatibility (re-passivation layer formed). This is a key factor in the long-term performance of implants [40]. The outcomes obtained create a new implant surface, achieving an area average roughness (Sa) value of ~0.5 µm (Figure 2e), comparable to that of machined implants, which reflects its smoothness. A measured binding energy of 459 eV confirms the presence of a passivation layer, demonstrating its stability and effectiveness. A clinical case demonstrates this step being performed in the mouth (Figure 3); a controlled bone wound was created simultaneously with the precise implantoplasty, thereby triggering the natural bone healing process. Achieving the correct treatment involves turning the implant surface to provide both resistance to recurrent inflammation and optimal osseointegration performance. The surface roughness of Ra 3.5-06 μm has been clinically validated as an essential step in peri-implantitis management.

**Figure 4:**
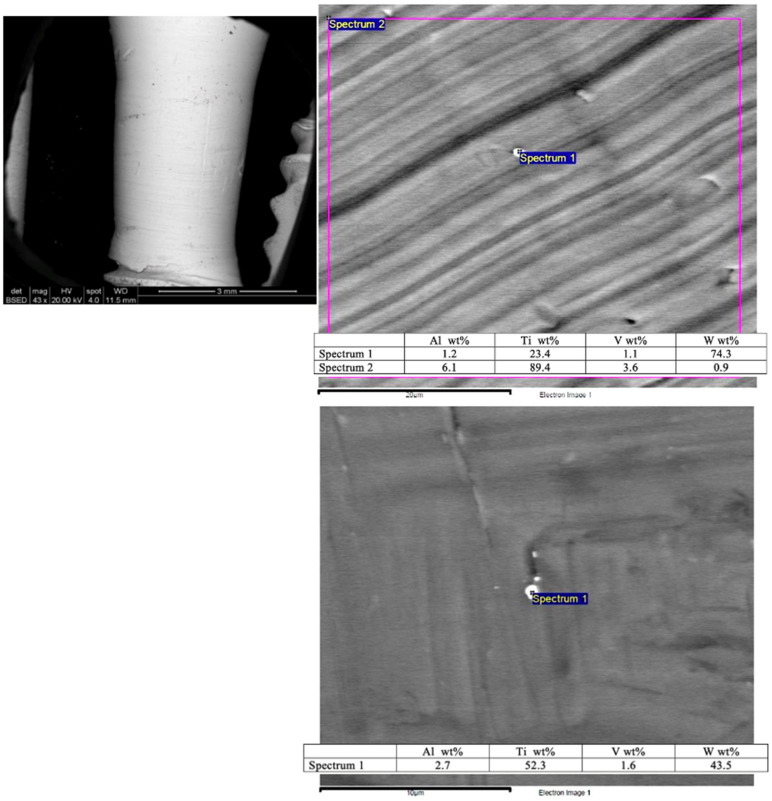
SEM and EDS analysis displaying the dental implant surface after treatment. The tests were conducted in triplicate.

A scanning electron microscopy (SEM) image shows the smooth surface that results from applying the Quadrant protocol with the iMPACT tool (Figure 4). Different areas presented minimal particles (<1 μm) on the implant surface, with superficial scratches resulting from the machining technique. Energy Dispersive X-ray Spectroscopy (EDS) analyses of these particles (Figure 4) reveal a high content of titanium (Ti) and tungsten (W) from the machining process tools, which is a residue from the microparticles. To classify the treated implants, both ISO 14607:2018 [45] and ISO ISO 25178-2:2021 [46] were used to support the measurement of surface roughness.

Step 2. Natural bone healing: Bone healing is critical in dental implant rehabilitation. This step facilitates the dynamic and coordinated biological processes necessary for bone regeneration and re-osseointegration. A controlled circumferential cut, performed in step 1, creates a precise 1.3 mm circumferential cut at the base of the defect, aligning it with the restored implant surface and establishing a conducive environment for bone healing. The hydrophilic nature of the new implant surface facilitates blood clot formation, which serves as the foundation for subsequent osseointegration. This promotes improved cellular responses at the bone–implant interface [47,48].

Step 3. Prohibiting recurrent infection: Preventing infection recurrence is paramount for the longevity of dental implants. This step minimizes bacterial adhesion and reduces the likelihood of reinfection. The infection prevention measures include (1) producing a smooth surface design, as the surface roughness (Sa ~0.6 µm, Figure 4) must be optimized to balance osseointegration and bacterial resistance. A smoother implant surface reduces bacterial colonization, while promoting favorable conditions for soft tissue integration [40]. (2) The hydrophilic nature of the surface further discourages bacterial adhesion, thereby enhancing soft tissue integration.

**Figure 5:**
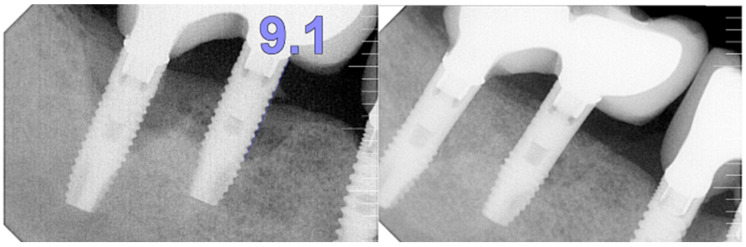
Periapical radiographies showing the re-osseointegration after using iMPACT and the Quadrant protocol. From the platform to the bone-implant contact (BIC), 9.1 mm was found.

Step 4: Re-osseointegration: This final step integrates the implant into the surrounding bone through orchestrated biological events. Completing the first three steps ensures the implant is biologically and structurally prepared for successful re-osseointegration. The hydrophilic surface promotes interaction between osteogenic cells and the implant, accelerating the re-integration process. Establishing a stable interface between the implant and surrounding bone ensures long-term success (Figure 5).

The dental implant surface undergoes in situ renewal, with surface quality as the primary focus, maintaining a surface roughness of less than 0.6 µm (machined). The new surface, hydrophilic, enhances tissue attachment and healing. An oxidation environment and passivation layer formation were achieved by ensuring consistent suction airflow around the prepared implant, thereby avoiding liquid cooling and strengthening the surface passivation layer [49].

### 4.1. Case Report 1

**Figure 6:**
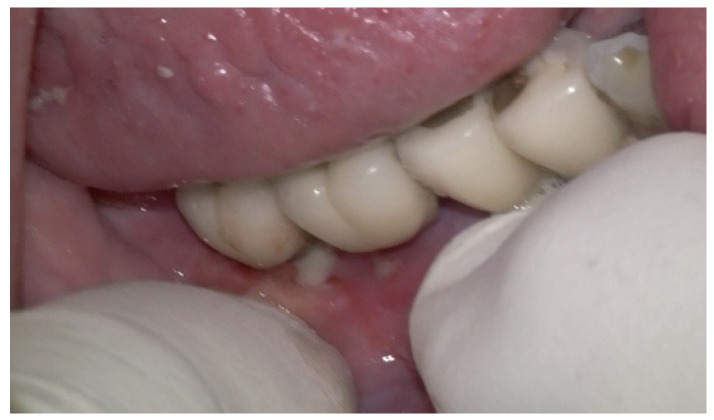
Initial clinical assessment of sites #46 and #47.

A 75-year-old male patient presented to the private clinic (Tel Aviv-Yafo, Israel) with severe pain, swelling, bad odor, and suppuration localized in the lower right posterior region (Figure 6). The chief complaint indicated discomfort and functional impairment related to that site. The implants placed in the first and second lower right molar region presented severe and vertical bone loss. The patient was a heavy smoker, 2–3 packs/day for 50 years, but quit smoking 5 years ago. Systemically, the patient had diabetes mellitus, controlled with daily insulin (HbA1C between 6% and 7%); osteoporosis, managed with bisphosphonates (via oral, around 2 years); and a cardiovascular condition, requiring anticoagulant therapy (more than 5 years).

**Figure 7:**
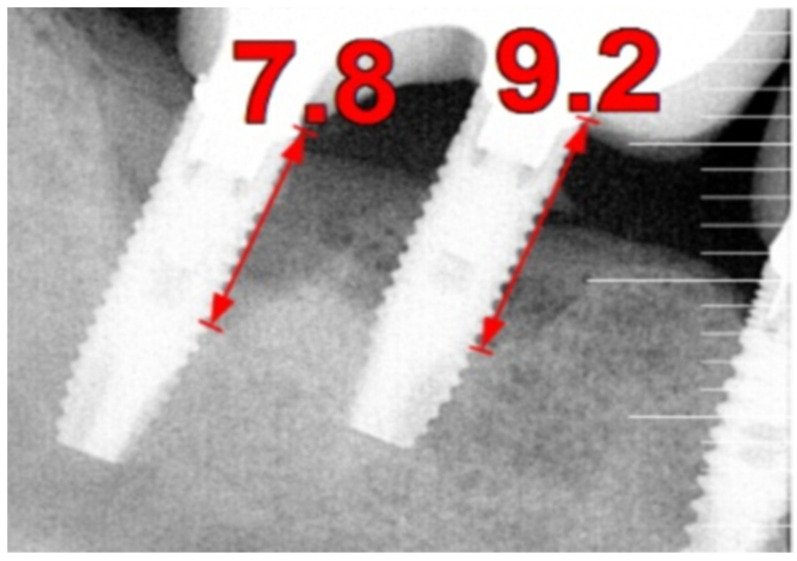
The periapical image taken for #46 and #47 (region of the complaint) shows relevant bone loss around the implants (red arrows showing the bone loss found, considering as reference the implant platform).

The intraoral examination revealed poor oral hygiene, characterized by significant plaque accumulation (over 65% plaque index) along the gingival margins and interdental areas. The periodontal status evidenced a progressive periodontal disease, with bleeding on probing (BoP) over 60%, mainly around all dental implants; peri-implant tissues possessed a high level of inflammatory condition, with localized swelling, high level of BoP, redness, and suppuration at the site of teeth #46 and #47, indicating local acute inflammation. The probing depth (PD) at site implant #46 exceeded 9 mm at both the mesial and distal sites, and at #47, the mesial site had a PD of 7.8mm, while the distal site showed lower bone loss (Figure 7).

**Figure 8:**
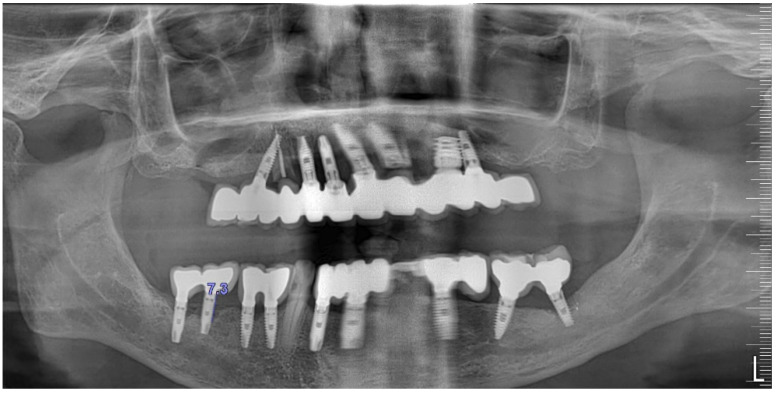
The initial panoramic radiograph reveals oral rehabilitation and bone loss in the lower posterior teeth.

Radiographically (Figure 8), the findings demonstrated full mouth edentulism, rehabilitated with upper and lower bridges and crowns supported by dental implants, as well as bone loss at #37, #46, and #47. The peri-apical radiograph (Figure 7) showed two dental implants, each measuring 13 mm, with bone loss around implant #46 (exceeding 60% of the implant length) and around implant #47 (exceeding 70% of the implant length).

Following consultation, treatment alternatives were proposed. As the site did not involve an aesthetic zone, the first option was to treat the implants with implantoplasty (applying a new tool [iMPACT^®^]), associated with bone graft; the second option was to retrieve the dental implants (#46 and #47), followed by guided bone regeneration (GBR) and future implant placement or just keeping a second pre-molar occlusion (lower right side). A decision was made to proceed with a surgical intervention with regenerative allograft and xenograft bone to manage peri-implantitis.

The preoperative management followed an antibiotic therapy, with a prescription of Augmentin^®^ 875 mg (GlaxoSmithKline (GSK), London, UK), taken twice daily for 8 days (starting 3 days prior to the surgical intervention), and anti-inflammatory medication, Ibuprofen 600 mg (Pfizer, Sandwich, UK), taken twice daily for 5 days; management of anticoagulation, requesting clearance and guidance from the cardiologist on the temporary cessation of anticoagulants.

**Figure 9:**
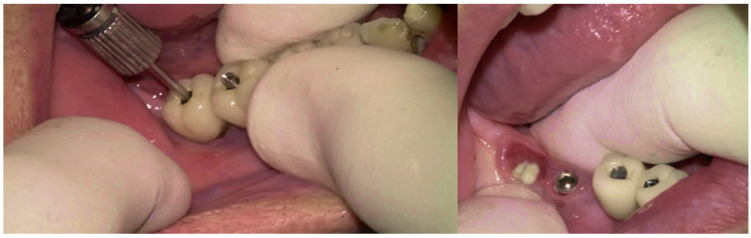
Removing the screwed crowns and for the site evaluation.

The surgical step began with local anesthesia (Lidocaine 2% with 1:100,000 epinephrine—Henry Schein Inc., New York, NY, USA) locally administered; the screw-retained crowns on the implants were removed to facilitate proper surgical access (Figure 9). The surgical site was assessed in order to provide a minimally invasive buccal flap design, which was favorable in this case, as it effectively executed the Quadrant protocol.

**Figure 10:**
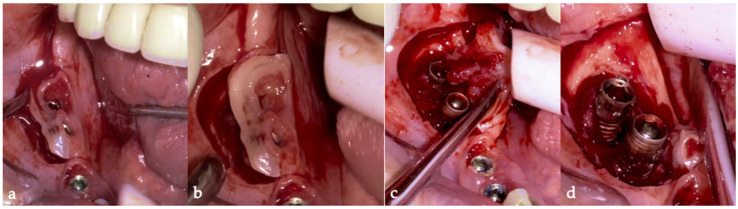
(**a**) BSF incision; (**b**) BSF raised; (**c**) BSF moved from buccal to lingual; (**d**) BSF raised exposing the implants and local bone.

The choice of flap design was crucial for optimal healing and patient outcomes. A buccal single flap (BSF) approach offers distinct advantages over the traditional midcrestal flap technique; BSF involves a precise incision on the buccal side, primarily targeting healthy tissue. This method enhances the visibility of the surgical area and minimizes trauma to inflamed tissues, facilitating easier suturing and manipulation. BSF permits better healing and reduces postoperative complications by avoiding incisions through fragile and inflamed tissues. Additionally, preserving the periosteal blood supply in this approach may minimize the loss of crestal ridge width and height, contributing to improved surgical outcomes. BSF design also reduces the injury to the keratinized tissue [50]. The advantages of the BSF are the reduced tissue trauma by limiting incisions to healthy tissue, minimizing damage to inflamed areas, leading to better healing and patient comfort; enhanced surgical visibility, providing a clear view of the surgical site, allowing for precise manipulation and suturing; the preservation of blood supply, maintaining the integrity of the periosteal blood supply, which supports bone preservation and reduces the risk of crestal bone loss [51] (Figure 10a,b).

**Figure 11:**
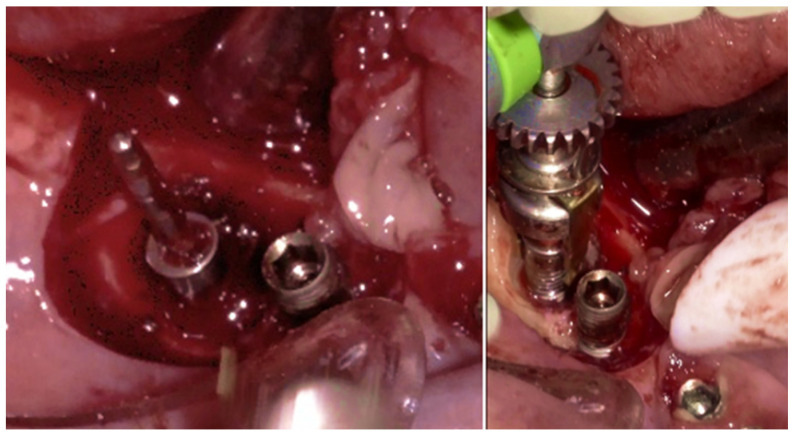
Quadrant protocol’s steps for the novel iMPACT tool application.

Then, the BSF was reflected to the lingual side (Figure 10c,d), exposing the surgical and infected site. Significant calculus deposits and plaque were noted on and around the implant surfaces. Then, with the screwed pin (hinge) in position, the implantoplasty was performed through the Quadrant protocol using the novel iMPACT device to renew the implant surface, transform it into a machined surface, and contour the surrounding bone (Figure 11).

**Figure 12:**
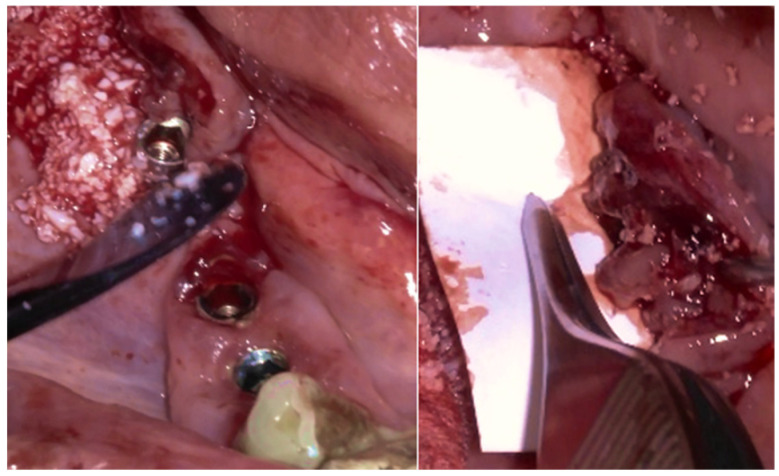
The bone graft (allograft + xenograft) is in place, and the collagen membrane covers the bone graft for the GBR.

**Figure 13:**
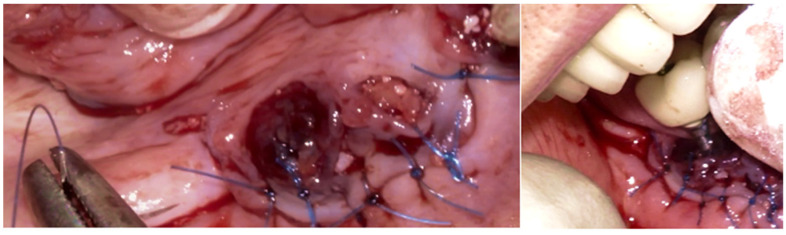
Interrupted suture and bridge reattachment, ensuring surgical site protection and occlusal function.

After completely smoothing the surface of the implants, guided bone regeneration (GBR) was applied (Figure 12); the grafting approach used a combination of 30% Raptos^®^ allograft (cancellous and cortical bone) (Citagenix, Laval, QC, Canada) and 70% bovine xenograft (Bio-Oss, Geistlich, Wolhusen, Switzerland) to maximize structural stability and bioactivity. The grafting material was inserted after being hydrated and condensed into the defect site. A resorbable collagen membrane (Bio-Gide, Geistlich, Wolhusen, Switzerland) covered the graft after it was positioned at the site. The site was sutured with Polyamide 4/0 (Ethicon Ethilon Nylon Suture, Raritan, Raritan, NJ, USA) at 1 mm intervals to ensure wound stability and optimal healing (Figure 13). After being cleansed, the prosthesis was adapted to fit the new gingival line, which changed due to the bone augmentation and suturing of the surgical site.

**Figure 14:**
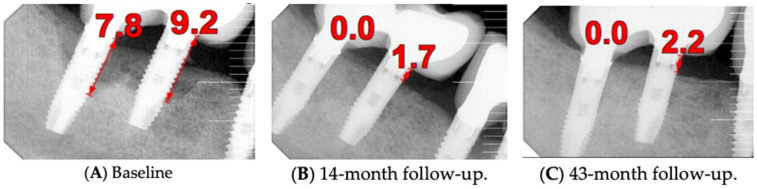
Periapical images showing the evolution of the case (red arrows show the distance from the implant platform to the bone—(**A**) Baseline; (**B**) After 14 months; (**C**) After 43 months).

**Figure 15:**
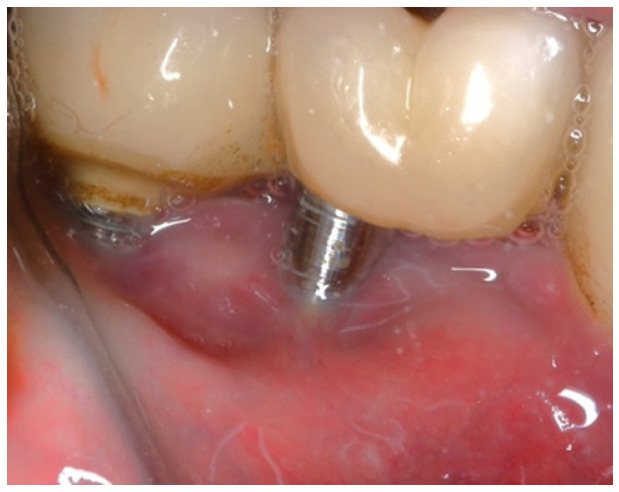
Clinical findings after 43 months.

The patient was provided with postoperative care instructions, including continuing with Moxypen 500 mg (Teva Pharmaceuticals, Petah Tikva, Israel) taken three times per day for three days, in addition to the medication previously prescribed. Follow-up appointments to monitor healing and treatment efficacy. The prosthesis adaptation to the gingiva was followed up for three months, when tissue healing was complete, and the need for readapting the prosthesis was evaluated. Considerations for readaptation included facilitating patient cleaning procedures. The patient was advised to enhance oral hygiene practices and scheduled for follow-up appointments. At the 14-month follow-up, the patient exhibited significant clinical improvements, with reduced PDs and evidence of bone regeneration around the affected implants (Figure 14). Notably, despite suboptimal oral hygiene practices being observed after 46 months, the patient maintained stable peri-implant conditions with minimal signs of mucositis, as evidenced by probing depths of 2 mm at implant #46 and 1 mm at implant #47, revealing significant improvements. This outcome underscores the potential durability of the surgical and regenerative interventions employed. However, it also highlights the critical importance of patient compliance with oral hygiene and regular maintenance visits in sustaining treatment outcomes, emphasizing the need for ongoing supportive peri-implant therapy. These findings suggest sustained peri-implant health over the long term. At the last follow-up, the patient reported being comfortable with the prosthesis and experiencing ease of cleaning. The intraoral X-rays indicated vertical bone gains of 7.5 mm around implant #46 and 8 mm around implant #47. Similar results were found after 43 months (Figure 14 and Figure 15), demonstrating the success of the treatment.

### 4.2. Case Report 2

**Figure 16:**
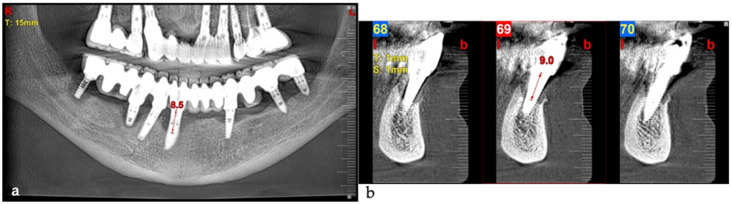
Panoramic radiographic view (**a**) and cone-beam computed tomography (CBCT) (**b**), revealing bone loss at baseline at tooth #41, respectively, 8.5 mm and 9.0 mm (red arrows).

An 80-year-old male patient presented at the private dental office with severe mandibular pain, swelling, and mobility of a dental prosthesis retained by implants. The chief complaint was the lack of stability and mobility in mandible rehabilitation, with the symptoms already described. The medical history included controlled hypertension, hypercholesterolemia, anticoagulant therapy, and a history of heavy tobacco use, which ceased 15 years prior to the oral rehabilitation. The clinical examination revealed inadequate oral hygiene, extensive fixed-prosthesis and dental implant mobility, with PDs exceeding 10 mm; significant BoP was found, and severe vertical bone loss was radiographically confirmed around implant #33, #36, #37, #41, #43, #44, and #47 (Figure 16).

**Figure 17:**
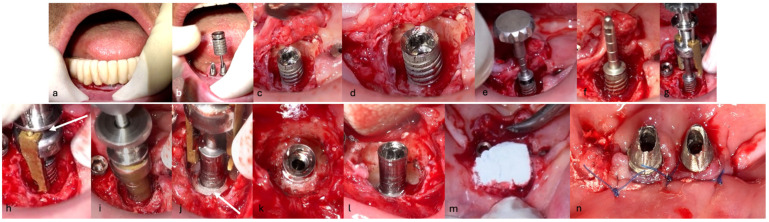
Step-by-step treatment for peri-implantitis using the iMPACT and Quadrant protocol. (**a**) Initial clinical evaluation; (**b**) Removing the abutments for peri-implantitis treatment; (**c**,**d**) BSF raised for implant exposition; (**e**) Insertion of the hinge (pin), which was crewed into the implant; (**f**) Hinge in position; (**g**,**h**) iMPACT adapted to the hinge; (**i**) iMPACT spinning for implantoplasty; (**j**) The implant surface was smoothed, and bone around the implant was gently cut (osteotomy); (**k**) occlusal view showing the osteotomy; (**l**) implantoplasty finished—implant surface is completely smoothed (machined); (**m**) Bone graft; (**n**) Suture and abutments were repositioned.

The same protocol mentioned above was used. A five-day antibiotic regimen was administered prior to the surgical procedure. At the surgery, the site had no suppuration and minimal BoP; the implants #33, #36, #37, #44, and #47 were removed, and new implants were placed to provide a new rehabilitation. For the site #41 (without mobility and severe bone loss), following prosthesis and abutment removal (Figure 17a,b), a BSF with two crestal extensions was made around the implants (Figure 17c,d), facilitating optimal access and flap management during the surgical procedure, eliminating the need to retract two flaps, as a midcrestal flap requires. The flap was retracted lingually; the granulation tissue was removed, and the contaminated implant surface fully exposed (Figure 17d).

The specialized “pin” guide was attached to the implant and contacts in juxtaposition with the surface of the implant–abutment connection platform (Figure 17e,f). The guide, extending the long axis of the implant, was used to position and control the iMPACT device’s rotational movement (Figure 17g,h), with cutting blades extended to maximum capacity (Figure 17h,i). Initial rotation levels of the alveolar bone (Figure 17j,k) permitted controlled mechanical injury to the bone, initiating the bone healing response, known as “Bone Regeneration Induction,” which is the second principle of the “Quadrant protocol.” Incremental machining at speeds between 200–300 RPM with torque settings of 7–9 N·cm removed contaminated implant threads (Figure 17l). The cutting action of the iMPACT prevents heating during manipulation, and the suction airflow removes macroparticles without the need for irrigation; thus, producing macro-sized particles removed via suction. The non-irrigation approach established an oxidative environment conducive to passivation layer formation. Post-machining (Figure 17l), the site was thoroughly cleaned and dried, and bleeding was stimulated and associated with the bone particulate graft (Figure 17m). The flap was repositioned, and abutments were placed back to keep and hold it in position for the final suture (Figure 17n). Antibiotics were continued postoperatively for three additional days.

**Figure 18:**
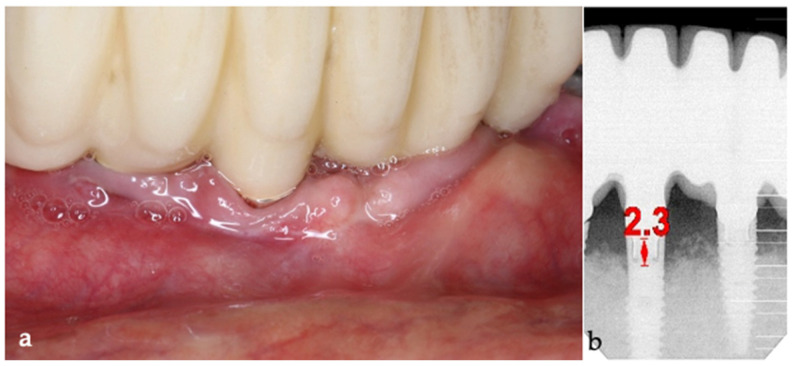
12-month follow-up (#41).

The three-month follow-up, with a new fixed rehabilitation, revealed healthy gingiva, the absence of BoP, significantly reduced probing depths (1 mm), and vertical bone gain of over 6 mm. The twelve-month follow-up showed continued gingival health, no BoP, PDs at 0–1 mm at tooth #41, and sustained bone gain totaling 6.7 mm, reflecting persistent osseointegration (Figure 18).

### 4.3. Case Report 3

**Figure 19:**
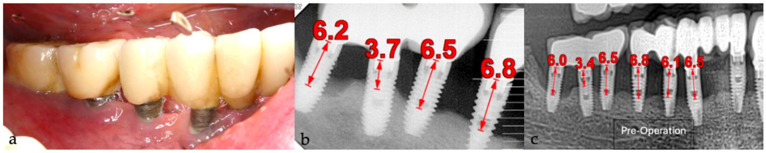
(**a**) Initial clinical assessment; (**b**) Periapical X-ray presenting the measurements (red arrows) from the platform to the bone; (**c**) The panoramic view shows the vertical bone loss found at baseline.

A generally healthy 63-year-old female presented to our clinic with chief complaints of foul odor and occasional pain emanating from dental implants located in the mandibular right quadrant (from implants #42 to #47). Clinical examination revealed peri-implantitis, with a high level of BoP and suppuration around the affected implants. The radiographic evaluation confirmed moderate-to-severe vertical bone loss, consistent with a diagnosis of peri-implantitis (Figure 19).

**Figure 20:**
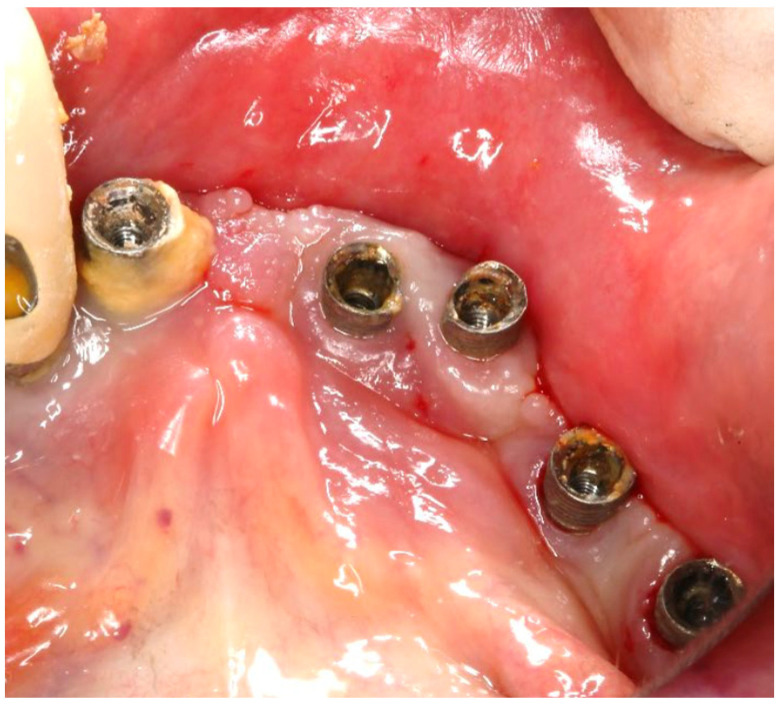
Fourth-quadrant condition after fixed prosthesis removal.

**Figure 21:**
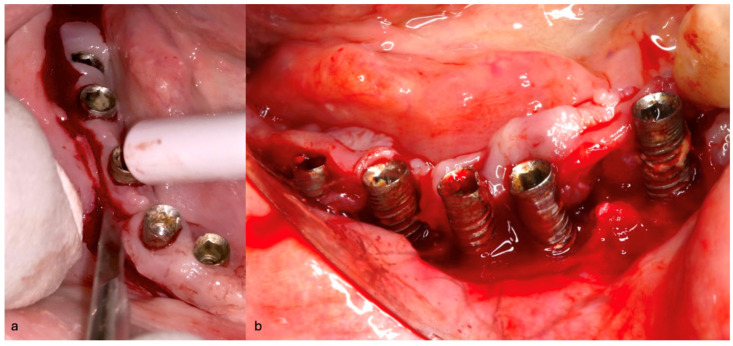
(**a**) BSF performed; (**b**) BSF raised and reflected to the lingual side, exposing the implants.

Before the surgical management following the Quadrant protocol, the fixed prosthesis was removed (Figure 20), and the region was cleaned. The surgical procedure involved a BSF, which was reflected lingually (Figure 21a,b) to facilitate access to the affected implant surfaces. With optimal access, thorough removal of granulation tissue occurred, minimizing the risk of lingual nerve damage and allowing for control of the flap.

**Figure 22:**
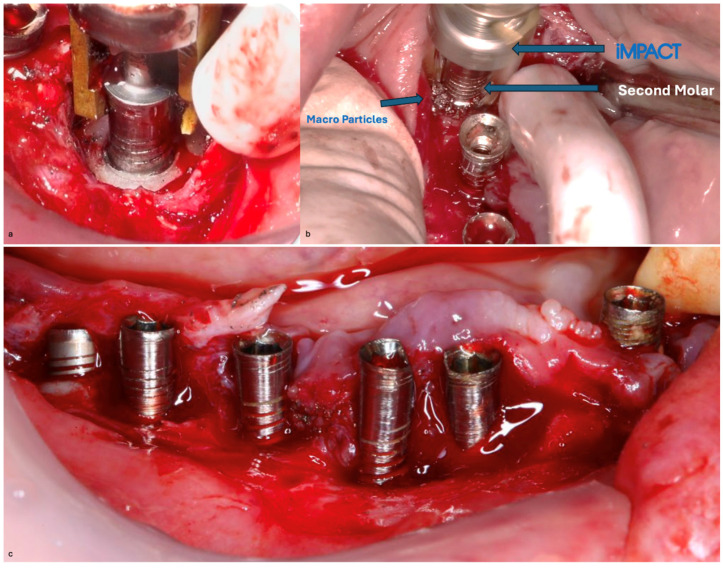
(**a**) iMPACT tool was placed for implantoplasty; (**b**) iMPACT spinning to remove the contaminated threads, machining the implant surface; (**c**) Result obtained after treatment.

The Quadrant protocol for peri-implantitis treatment was applied, as previously described. The dental implants #42–#47 received the insertion of the iMPACT to remove residual biofilm and contaminants directly from the implant surface, effectively renewing them without the need for implant retrieval. This step revitalized the implant surface, eliminating factors that contribute to peri-implantitis and establishing a stable foundation for healing (Figure 22a–c). The iMPACT’s cutting blades cut the implant surface and a portion of the bone (a controlled procedure). The granulation tissue, residual biofilm, and contaminants were meticulously removed with the tool, facilitating the restoration of implant surface integrity.

**Figure 23:**
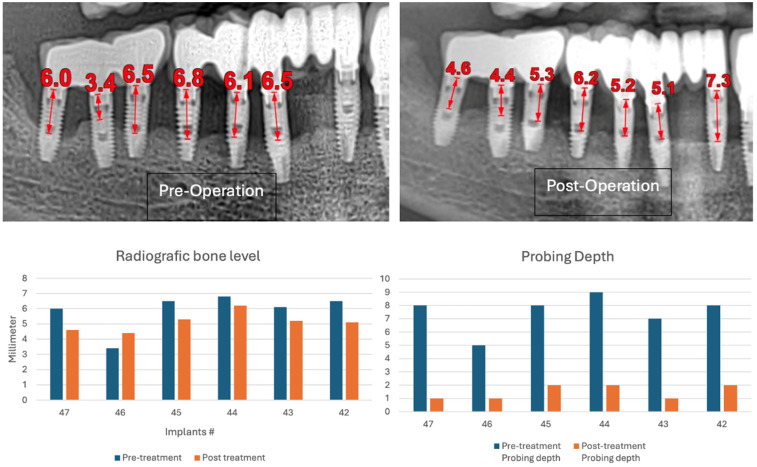
Radiographic bone level and probing depth comparing the baseline and 12-month results.

**Figure 24:**
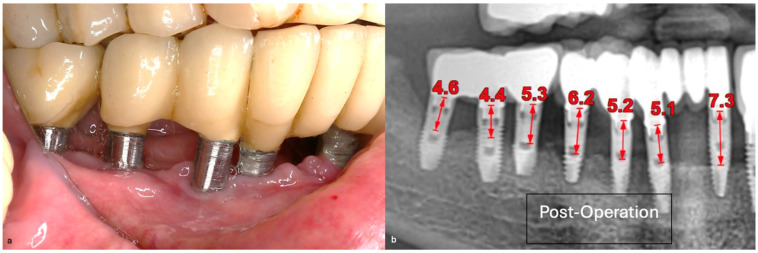
(**a**) Clinical follow-up after 12 months; (**b**) Radiographic outcome after 12 months.

In this case, after achieving the renewed implant surface (machined), the regenerative procedure involving bone grafting and collagen membrane was not performed. The flaps were repositioned and secured with tension-free primary closure. The postoperative instructions included chlorhexidine 0.12% (Taro Pharmaceuticals, Haifa Bay, Israel) rinses and systemic antibiotic coverage for 5 days. Regular clinical and radiographic evaluations were conducted at 1 month, 3 months, 6 months, and 12 months, postoperatively. At the six-month follow-up, the clinical examination revealed significant improvements, with resolution of inflammation and no BoP; the patient reported a complete resolution of the initial chief complaints. Radiographs demonstrated stabilization and initial signs of bone regeneration around treated implants. At the 12-month follow-up, the bone level and PDs were stabilized (Figure 23), and the implant surface adequately accommodated the gingival tissue (Figure 24).

## 5. Discussion

This article aimed to introduce, for the first time in the literature, a new device (iMPACT) for treating peri-implantitis, providing a more standardized implantoplasty technique based on a new Quadrant protocol. In the in vitro evaluation, SEM images and EDS verified a machined and highly smooth surface with minimal microparticle remnants, resulting from the controlled implantoplasty process. Del Amo et al. [31] observed that Ti particles are a common finding surrounding peri-implant tissues, particularly in PI sites, with a higher concentration of particles than in healthy implants. This scenario can be minimized using the standardized Quadrant protocol, as the production of macroparticles in the majority is easily suctioned and cleaned from the sites, as presented in the reported cases. Free Ti particles/debris detached from the implant surface upon placement or during the implantoplasty procedure may trigger a deoxyribonucleic acid (DNA) damage response signaling in oral epithelial cells, suggesting their contribution to the disruption of epithelial homeostasis and potentially compromising the oral epithelial barrier [52].

Thus, it is possible to affirm that implant surfaces impact the biological local response; hydrophilicity, nanotexturization, multifunctional coatings, and incorporated drug-release systems have demonstrated favorable outcomes [53]. However, in cases of peri-implantitis, these surfaces have a higher potential for contamination, thereby increasing the local inflammatory burden and bacterial accumulation [54]. This fact is reduced if the Quadrant protocol is applied using iMPACT; first, because it involves the complete removal of contaminated threads, and the resulting machined and smooth surface reduces the chance of recontamination, as demonstrated in the reported cases. Hence, Kunrath et al. [53] concluded that emerging approaches in surface modifications, functionalized with epigenetics [44], have great potential with a significant impact on modulating bone healing during osseointegration.

As Martins et al. [14] described in an umbrella review, comparing non-surgical and surgical approaches to treat PI, surgical treatment is the most efficient, and implantoplasty is the most significant surgical technique, yielding better outcomes. Within this context, iMPACT has been used to treat PI cases. The three cases presented using the Quadrant protocol with iMPACT showed significant clinical PI resolution. Moreover, in cases where a bone graft was used, combining a resective approach with a regenerative procedure, it was feasible to detect the re-osseointegration of the new, intra-oral, machined implant surface. This fact was also stimulated in all cases presented due to the small osteotomy around the implants performed using iMPACT. Madi et al. [55], in a systematic review article, presented promising results combining bone substitutes and GBR, similar to two cases from the present study. However, on the other hand, they considered the implant surface with a better re-osseointegration level to be the rough implant surface rather than the smooth surface. Given the small number of articles included (n = 15), the diverse methodologies applied without statistical treatment (meta-analysis), and the absence of a quality assessment, these results should be carefully interpreted. Schwarz et al. [56] evaluated 17 patients after 48 months who received two different surface decontamination methods with regeneration (Er:YAG laser or plastic curettes + cotton pellets + sterile saline). The authors concluded that resective/regenerative therapy of advanced peri-implantitis was not influenced by the method applied.

Regarding the clinical approach, a comprehensive assessment highlights several critical factors that contribute to successful peri-implantitis management. Professional experience should be considered; also, patients with general health and no smoking or smoking cessation for at least one year can positively impact peri-implant healing after treatment. Furthermore, managing hypertension, cholesterol levels, and other possible systemic conditions effectively supports optimal tissue healing. Adequate oral hygiene should be maintained, which impacts the plaque index and BoP, particularly post-procedure, and contributes significantly to tissue stability. The surgical approach, utilizing the Quadrant protocol and iMPACT device, facilitated minimally invasive and effective implant renewal, bone regeneration, and the prevention of microbial contamination. It is worth noting that the attempt to control the oxidative environment created by the iMPACT device machining process may have significantly contributed to the favorable outcomes, including the adherence and osseointegration of bone cells [57]; this fact warrants further investigation. Outcomes presented in this study were robust, with sustained clinical improvements in the medium/long term, reinforcing the effectiveness of the Quadrant protocol. The comprehensive integration of this protocol and tool suggests a promising future for minimally invasive treatments in cases of moderate-to-severe peri-implantitis. Moreover, the decision to use a resective treatment approach in the current cases presented was based on the lack of/minimal aesthetic concerns. This allowed for a simplified surgical approach, ensuring medium/long-term implant stability. The iMPACT device played a crucial role in decontaminating and modifying the implant surface, creating a predictable and bacteria-resistant environment with smoother surfaces and a conductive surface for new tissue deposition and accommodation.

As a limitation of this study, the number of patients included can be considered low (3 case reports), with two presenting outcomes in medium-term follow-up. Moreover, there was a lack of comparison between the GBR and non-GBR approaches, hampering the verification of the effectiveness of the regenerative procedure, as well as the absence of comparative cases using another type of approach. Also, it is highly recommended to be careful in this type of approach (implantoplasty), even though iMPACT with the Quadrant protocol is the most standardized approach (reducing the wear level of the implant structure), due to the high level of complexity, suggesting the presentation to the patient of treatment alternatives and balancing the surgeon’s ability and experience. For future studies, more patients should be included, the follow-up assessment should be increased, as demonstrated by one of the cases of this study (43 months), the inclusion of a comparative group using another technique to treat PI, and the evaluation of the inflammatory level (biomarkers), which was not performed in the present study. In addition, attention should be given to the keratinized tissue width around implants, as observed by Silva et al. [58], which can play a crucial role in the prognosis of peri-implantitis.

## 6. Conclusions

Within the limitations of this study, an innovative device and protocol were presented to treat PI cases, namely iMPACT and the Quadrant protocol, which achieved favorable outcomes in the three clinical cases, with re-osseointegration when combined with regenerative procedures. Thus, it was possible to reject the null hypothesis. The successful management of advanced PI should involve a comprehensive approach to surgical intervention (implantoplasty), implant surface decontamination, and regenerative techniques. The favorable medium/long-term outcomes achieved, despite the patient’s complex medical history and, at times, inconsistent oral hygiene, underscore the potential efficacy of such interventions. Nonetheless, the cases also highlight the importance of patient adherence to maintenance protocols in preventing disease recurrence. Further research, employing a strong methodology, such as RCTs, is recommended to compare the protocol of this study with that of another type of implantoplasty technique. This comparison is necessary to optimize treatment strategies and identify predictors of long-term success in peri-implantitis management.

## Data Availability

All data were included in the article.
